# Using Network Component Analysis to Dissect Regulatory Networks Mediated by Transcription Factors in Yeast

**DOI:** 10.1371/journal.pcbi.1000311

**Published:** 2009-03-20

**Authors:** Chun Ye, Simon J. Galbraith, James C. Liao, Eleazar Eskin

**Affiliations:** 1 Bioinformatics Program, University of California San Diego, La Jolla, California, United States of America; 2 Department of Computer Science, University of California Los Angeles, Los Angeles, California, United States of America; 3 Department of Chemical and Biomolecular Engineering, University of California Los Angeles, Los Angeles, California, United States of America; 4 Department of Human Genetics, University of California Los Angeles, Los Angeles, California, United States of America; University of Auckland, New Zealand

## Abstract

Understanding the relationship between genetic variation and gene expression is a central question in genetics. With the availability of data from high-throughput technologies such as ChIP-Chip, expression, and genotyping arrays, we can begin to not only identify associations but to understand how genetic variations perturb the underlying transcription regulatory networks to induce differential gene expression. In this study, we describe a simple model of transcription regulation where the expression of a gene is completely characterized by two properties: the concentrations and promoter affinities of active transcription factors. We devise a method that extends Network Component Analysis (NCA) to determine how genetic variations in the form of single nucleotide polymorphisms (SNPs) perturb these two properties. Applying our method to a segregating population of *Saccharomyces cerevisiae*, we found statistically significant examples of *trans*-acting SNPs located in regulatory hotspots that perturb transcription factor concentrations and affinities for target promoters to cause global differential expression and *cis*-acting genetic variations that perturb the promoter affinities of transcription factors on a single gene to cause local differential expression. Although many genetic variations linked to gene expressions have been identified, it is not clear how they perturb the underlying regulatory networks that govern gene expression. Our work begins to fill this void by showing that many genetic variations affect the concentrations of active transcription factors in a cell and their affinities for target promoters. Understanding the effects of these perturbations can help us to paint a more complete picture of the complex landscape of transcription regulation. The software package implementing the algorithms discussed in this work is available as a MATLAB package upon request.

## Author Summary

One of the fundamental challenges in biology in the post-genomics era is understanding the complex regulatory mechanisms that govern how genes are turned on and off. In a single organism where the functions of individual genes in a population do not differ much, many of the differences between individuals including physical phenotypes, susceptibility to disease, and response to drugs can be attributed to how genes are regulated. Previous studies have largely focused on identifying regulator and target genes whose expressions are linked to genetic variations in a population. We present work that focuses on considering a specific set of regulators called transcription factors whose targets can be verified from experiments and whose interactions with those targets have been well studied and modeled. In this setting, we can begin to understand how genetic variations perturb the concentrations and promoter affinities of active transcription factors to induce differential expression of the targets. Understanding the effects of these perturbations is important to understanding the fundamental biology of gene regulation and can help us to design and assess therapeutics and treatments for complex diseases.

## Introduction

With advances in whole genome high-throughput technologies such as ChIP-Chip, expression, and genotyping arrays, it is now possible to integrate data from these sources together to decipher the complex regulatory networks that govern transcription. In addition to serving as powerful models for how basic cellular function is achieved, these regulatory networks can also help us shed light on how certain disease phenotypes are manifested. At the heart of these networks are a few regulator genes such as transcription factors (TFs), miRNAs and histones whose activity govern the behavior of many other genes. Among these regulators, transcription factors that bind the promoter regions of genes are by far the most well understood. The process of TFs activating or repressing transcription at initiation is believed to be the primary mechanism of gene regulation. A central question in genetics is how genetic variations perturb this underlying regulatory mechanism to give rise to differential gene expression and ultimately complex phenotypes.

The simplest analysis one can perform to address this question is expression quantitative trait loci (eQTL) mapping, which identifies genetic variations such as SNPs in the form of linkages and associations that are correlated with gene expression. Such studies have been carried out in a variety of organisms including yeast [1],[2] Arabidopsis [3], mouse [4],[5] and human [6]–[8]. These studies have identified many linkages between SNPs and genes in close proximity suggesting potential local regulatory mechanisms mediated by regulators such as transcription factors and miRNAs. These studies have also identified a few SNPs linked to the expressions of many genes suggesting a global regulatory mechanism mediated by master regulators such as transcription factors and histones. Unfortunately, beyond nominating candidate genes either as targets or regulators, these studies give little insight into how SNPs perturb the underlying transcription regulatory networks that control gene expression.

To gain a better understanding of the mechanisms of transcription regulation, several systems biology based methods have been proposed including clustering of co-regulated genes [9], multipoint linkage analysis [10],[11], pathway enrichment analysis [12]–[16], prediction of regulatory modules [17],[18] and the prediction of causal regulatory relationships [19]–[23]. Many of these advanced methods aim to tease out both the nodes (regulators and targets) as well as the topology (mapping of edges) in a transcription regulatory network from only considering gene expression profiles. Although these methods have predicted some interesting relationships, there are at least two aspects of transcription regulation that go unaddressed when we use them to study transcription factors and their targets. First, most previous methods rely on probabilistic models that do not provide much insight into the hidden dynamics between the activity of transcription factors and the expression of their targets. Second, the relationships inferred by these methods from the expression profiles alone can be misleading because the *in vivo* activity of a transcription factor does not always correlate with its expression levels [24],[25].

To overcome these problems, we adopt a framework from network component analysis (NCA) [26] that considers a simple bipartite network model of transcription regulation involving only transcription factors and their targets. In this model, the expression of a target gene is completely captured by two properties of the network, the concentrations and promoter affinities of transcription factors. In general, inferring these two quantities from the expression profiles of the target genes alone is difficult. But by leveraging protein-DNA binding data from ChIP-Chip experiments [27],[28], a partial topology of the network can be constructed and one can make the inference given certain constraints [26].

**Figure 1:**
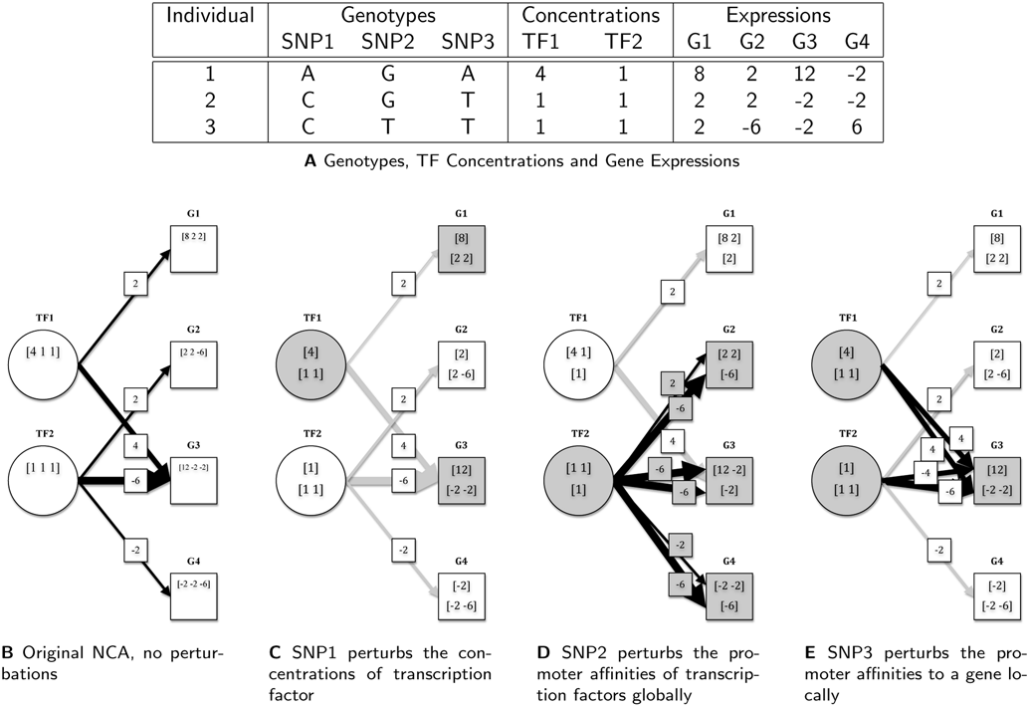
Graphical illustration of NCA and extension of NCA to include genetic perturbations. (A) A small toy example of three individuals with known genotyping and expression levels and inferred concentrations of active transcription factors. Each row corresponds to the genotypes, gene expressions and inferred transcription factor concentrations collected in one individual. (B) NCA regulatory network model when the network is unperturbed and the expression levels of G1, G2, G3 and G4 are determined by the concentrations of TF1, TF2 and the corresponding promoter affinities. (C) Between individuals with the A allele (1) and C allele (2,3) at SNP1, the concentrations of TF1 is perturbed by SNP1 causing differential expression of G1 and G3. (D) Between individuals with the G allele (1,2) and T allele (3) at SNP2, the promoter affinities of TF2 are perturbed globally by SNP2 (i.e. edges from TF2 are perturbed) to cause differential expression in all of TF2's targets G2, G3, and G4. (E) Between individuals with the A allele (1) and T allele (2,3) at SNP3, the affinities of TF1 and TF2 for the G3 promoter is perturbed locally by SNP3 to cause differential expression of G3.

The NCA method as described by liao et al. has been successfully applied to several gene expression datasets to understand transcription regulation in a temporal setting [26] and in the context of gene knockouts [29]. In this study, we extended NCA to study transcription regulation over a population gradient by modeling three mechanisms by which genetic variations perturb the concentrations and promoter affinities of active transcription factors to induce differential expression. Figure 1 gives a simple example that illustrates the original NCA model and our extensions. Imagine we have a small experiment where we collected the gene expressions of four genes, the genotypes of three markers over three individuals. Given the topology of the bipartite network between transcription factors and their targets (Figure 1B), the NCA algorithm allows us to infer the active transcription factor concentrations (C) and the respective promoter affinities (PA) from the given gene expressions (E) in a log-linear fashion (Figure 1A, see Methods). In this example, SNP1 and SNP3 are linked to the expressions of G1 and G3 while SNP2 is linked to the expressions of G2 and G4. We propose three possible mechanisms any one SNP can perturb the regulatory network and show an instance of each using the given example.

- **SNP perturbs the concentration of an active transcription factor.** SNP1 is linked to the concentration of TF1 and expressions of G1 and G3, both targets of TF1 (Figure 1C). Biologically, SNP1 could be located in close or far proximity to TF1 to change the concentration of TF1 *in vivo* through transcriptional, translational or post translational regulation causing differential expression of the target genes.
- **SNP perturbs the promoter affinities of a transcription factor globally.** SNP2 is linked to the expressions of G2 and G4, both targets of TF2. Here, SNP2 is not linked to the concentration of TF2 but can still mediate global differential expression by altering the promoter affinities of TF2 on its targets (Figure 1D). Biologically, SNP2 could be located either in close or far proximity to TF2 and alters TF2's affinities to many promoter regions either through a rare non-synonymous mutation or a change in binding affinity between transcription factors in a complex, causing the global differential expression of the target genes.
- **SNP perturbs the promoter affinities of transcription factors on a gene locally.** SNP3 is linked to the expression levels of G1 and G3 but is only *cis* to G3. It perturbs the local promoter affinities of TF1 and TF2 on G3 causing differential expression of G3 (Figure 1E). Biologically, SNP3 could be located in G3's promoter region altering the promoter affinities of a transcription factor (i.e. TF1) or a complex of transcription factors (i.e. TF1 and TF2), causing local differential expression of the target gene between populations. This mechanism differs from SNPs perturbing promoter affinities globally in that differential expression for only one gene (local), versus many genes (global) is induced.

Because the inclusion of genetic variation creates additional parameters in each of our three models compared to the original NCA model, we expected them to always fit the data better. To effectively evaluate our models, we devised a likelihood ratio statistic and a permutation scheme to assess the statistical significance of our improvements. We then applied our method to study an expression data collected over 112 segregants of *Saccharomyces cerevisiae* yeast and two separate ChIP-Chip datasets generated by Harbisonet al. and Lee et al..We identified several interesting global regulatory networks perturbed by SNPs located in regulatory hotspots. Some of these networks have one property perturbed (transcription factor concentration or promoter affinity) while others have both properties perturbed suggesting a complex mechanism of global regulation. We also examined linkages between SNPs and target genes located in close proximity. We found that many of these *cis* linked SNPs perturb the promoter affinities of transcription factors on a target gene locally confirming previous hypotheses of *cis* regulation.

An interesting method proposed by Sun et al. also used the NCA framework to infer the concentrations of active transcription factors from gene expression data collected over the same yeast strains. Their method was designed to detect linkages between the inferred concentrations and genetic variations and used conditional independence tests to find modules of genes controlled by the same causal regulator. Compared to this method, we expect to find similar networks of genes and transcription factors but our method does not allow us to infer additional causal relationships using statistical tests. Instead, we focus on identifying different mechanisms by which genetic variations can perturb the regulatory networks by directly modeling the effects of these perturbations into the NCA framework. We do not attempt to make rigorous causal claims but use the causal information inherent in genotyping and ChIP-Chip experiments to suggest possible mechanisms of transcription regulation.

## Results

### Inferring Concentrations and Promoter Affinities of Active Transcription Factors over a Population Gradient

**Figure 2:**
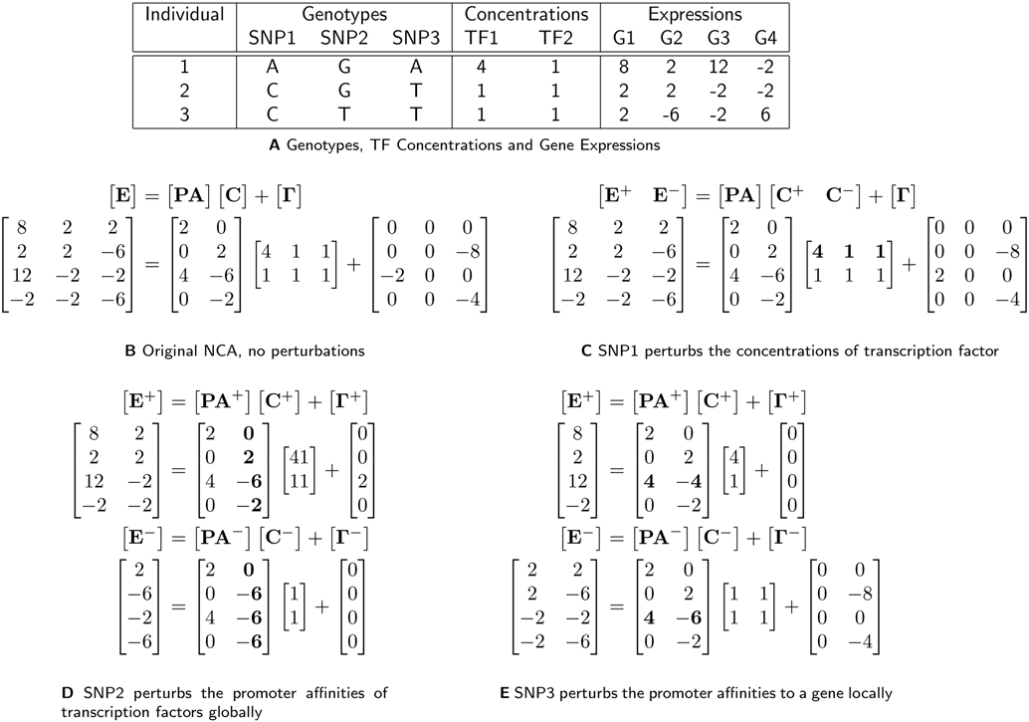
Matrix representation of NCA and extension of NCA to include genetic perturbations. (A) The same small toy example as Figure 1A. Log-linear equation representations of (B) the unperturbed NCA regulatory network model, (C) SNP1 perturbing the concentration of TF1, (D) SNP2 perturbing the promoter affinities of TF2 for its targets, (E) SNP3 perturbing the promoter affinities of TF1 and TF2 for G3.

The NCA framework is a natural model for describing how transcription factors regulate gene expression. At the heart of the model is a log linear equation that relates the expression levels of genes collected over a gradient (E) to the concentrations (C) and promoter affinities (PA) of active transcription factors. Such a model is well supported by known kinetic properties of protein-DNA interactions [30]. In linear model terms, the transcription factor concentrations are the regressors, the gene expression levels are the response variables and the promoter affinities are the coefficients that relate the two. Figure 2B shows the log-linear equations describing the graph shown in Figure 1B. The goal of NCA is to infer the matrices of concentrations 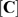 and promoter affinities 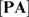 from the matrix of gene expressions 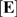 under some restrictions in the least squares sense.

Treating genetic differences between individuals as a gradient, we applied this model to infer the matrices 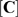 and 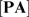 from gene expressions collected from a population of yeast strains, 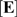. For the inference to have been possible, we removed a number of transcription factors and target genes to construct a network from the original ChIP-Chip data that met certain constraints [26]. After preprocessing the Lee et al. ChIP-Chip dataset, we were left with a network with 100 transcription factors and 2,294 target genes. Similarly, preprocessing the Harbison et al. ChIP-Chip dataset left 158 transcription factors and 2,779 target genes. Using a two step optimization algorithm developed by Liao et al., we inferred the concentration profile for each transcription factor over the genetic gradient and compared it to the corresponding TF expression profile by computing Pearson's correlations (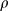). Figure S3 shows that these quantities were not well correlated with average correlation coefficients of 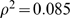 and 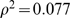 using the Lee et al. and Harbison et al. datasets respectively. The stability of the inferred TF concentrations were however robust when we compared results from the two ChIP-Chip datasets with a correlation coefficient of 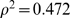 (Figure S4). The robustness was also verified by bootstrapping experiments [31] (Results not shown).

### Identifying Regulatory Hotspots

We next applied our method to study the mechanisms by which regulatory hotspots, genomic locations in yeast shown to be linked to the expression of many genes, perturb the underlying transcription regulatory networks. Although several transcription factors have been known to act as master regulators in yeast, it has been surprisingly shown in previous eQTL studies that only a few regulatory hotspots are located close to transcription factors. We hypothesized that although complex regulatory mechanisms upstream of transcription regulation such as signaling pathways exist, transcription factors ultimately mediate the global regulation of gene expressions. Using our framework, we tested our hypothesis by determining whether a regulatory hotspot is linked to the concentrations or promoter affinities of active transcription factors to achieving this regulation.

To identify the regulatory hotspots, we performed simple linkage analysis on only a subset of genes that were NCA compliant (see Methods). Similar to previous reports, only a few hotspots were located *cis* to any known transcription factors [1],[2]. For example, a hotspot located on chromosome 12 spanning basepairs 600,000 to 680,000 was *cis* to *HAP1* while another hotspot located on chromosome 3 spanning basepairs 60,000 to 100,000 was *cis* to *LEU3*. Several approaches [20],[23] have identified additional putative causal regulators, many of which are not transcription factors, corresponding to these regulatory hotspots.

### Regulatory Hotspots Perturbed the Concentration of Active Transcription Factors To Cause Global Differential Expression

We first considered SNPs located in regulatory hotspots that perturbed the concentrations of active transcription factors to cause global differential expression. Extending the NCA model to incorporate SNPs as perturbations did not require changing the optimization procedure. As shown in Figure 2C, we first decomposed the inferred transcription factor concentration matrix from applying the original NCA algorithm, 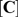, into two matrices 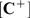 and 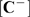 segregated by a SNP. Next, we identified those transcription factors whose concentrations were linked to the SNP using a simple *t*-test, an example is shown in bold in Figure 2C, and assessed the significance of the linkage by a permutation scheme (see Methods).

Using both the Harbison et al. and Lee et al. ChIP-Chip binding data, we found many transcription factors whose concentrations were linked to at least one SNP. Table 1 lists those linkages occurring at regulatory hotspots and the corresponding transcription factors. In addition to having a strong linkage, we also required the transcription factors in the table to have at least 6 (Lee et al) or 7 (Harbison et al) downstream targets whose expression levels were significantly linked to the regulatory hotspot. A number of transcription factors known to act as global regulators were identified. Of particular note, we found *HAP1* to be the mediator of hotspot 6 located on chromosome 12 spanning basepairs 600,000 to 680,000 using the Harbison et al. dataset; and *YAP1* and *LEU3* to be mediators of hotspot 3 located on chromosome 3 spanning basepairs 60,000 to 100,000. *GCN4* was also identified as a mediator of this hotspot using the Lee et al. dataset but it was only marginally significant using the Harbison et al. dataset (Result not shown). These results are concordant with previous findings [2],[23]. In particular, *LEU2* has been previously implicated to be linked to hotspot 3 where an engineered deletion of the gene occurs. Figure 4 are heatmaps showing the strong correlations between concentration levels of transcription factors, *HAP1* and *LEU3* respectively, and the expression levels of their downstream targets linked to the respective regulatory hotspots.

**Table 1:** Regulatory hotspots and the transcription factors whose active concentrations are perturbed to achieve global regulation.

	Hotspot Location			# Linkages		Significant TFs		
	Chr	Begin	End	Lee	Harbison	Lee	Harbison	Shared
1	2	360000	380000	24	29	None	None	FHL1
2	2	480000	580000	103	142	None	ABF1, FKH1, OAF1 RAP1, SWI5	ACE2, MBP1, SKN7 SWI4
3	3	60000	100000	89	113	GCN4, MCM1, MET4	MET32	LEU3, YAP1
4	5	340000	440000	34	48	None	SUT1	None
5	8	80000	120000	36	51	None	None	DIG1
6	12	600000	680000	54	91	None	HAP1	None
7	12	1040000	1060000	8	12	None	GAT3	YAP5
8	13	40000	60000	20	27	None	None	BAS1
9	14	440000	500000	130	179	None	None	None
10	15	140000	200000	76	117	HAL9, RAP1, SWI5	FKH2, NDD1	None
11	15	560000	580000	21	26	None	None	HAP4

**Figure 3:**
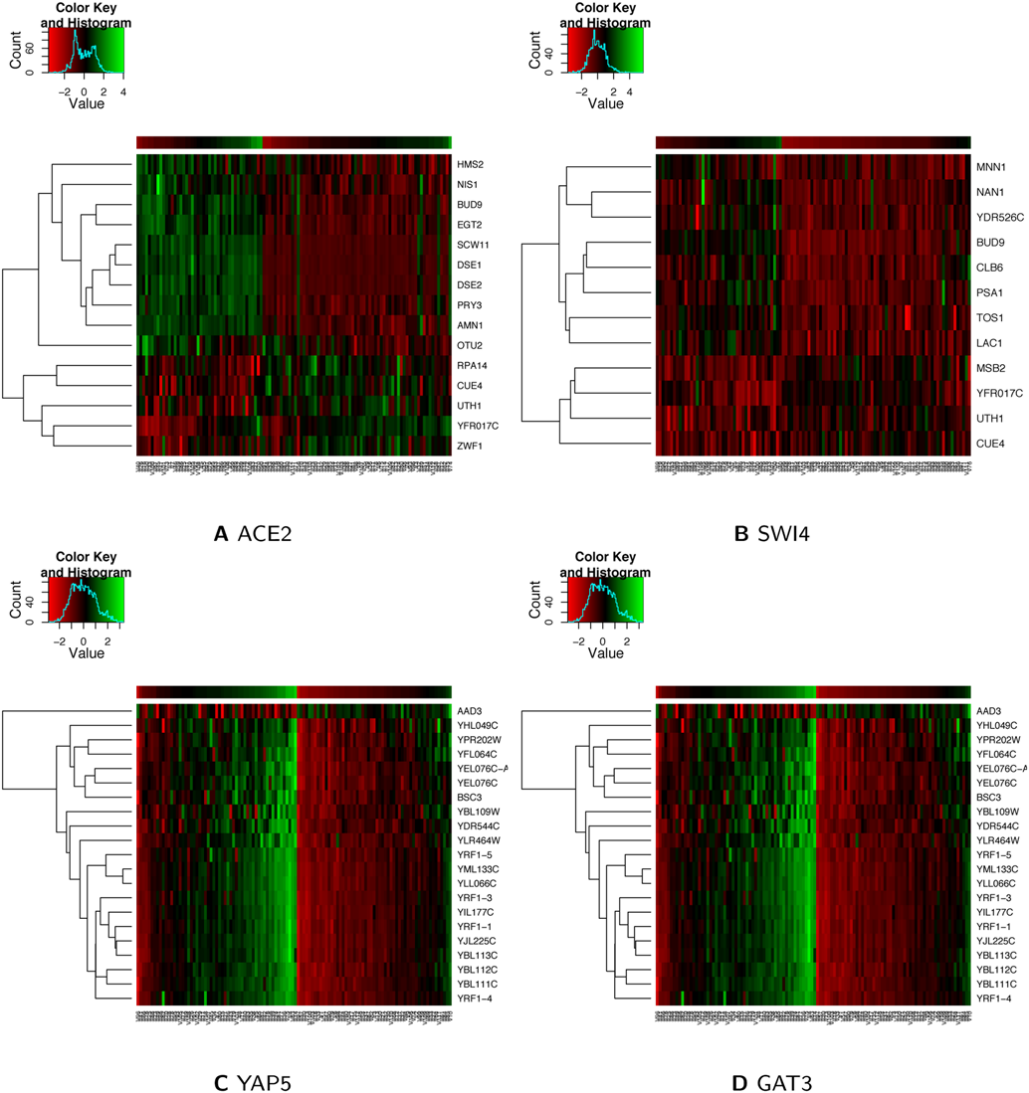
Correlations between concentrations of transcription factors and the expressions of their targets. Heatmap showing the correlations between concentrations of transcription factors and the expressions of their downstream targets linked to hotspot 2 on chromosome 2 ((A) *ACE2* and (B) *SWI4*) and hotspot 7 on chromosome 12 ((C) *YAP5* and (D) *GAT3*). The bar above each heatmap designates the concentration profile of each transcription factor.

We next examined hotspot 2, a hotspot that has been previously identified by brem et al.to regulate budding and daughter cell separation through the causal regulator *AMN1* [9]. We identified four transcription factors, *ACE2*, *MBP1*, *SKN7* and *SWI4*, whose active concentrations were significantly linked to hotspot 2 in both datasets. Five other transcription factors responsible for cell cycle transitions, *ABF1*, *FKH1*, *OAF1*, *RAP1* and *SWI5* were also found to be significant in the Harbison et al. dataset. Some of these transcription factors are known to interact with each other and have similar profiles such as *ACE2* and *SWI5*; and *MBP1*, *SKN7* and *RAP1*. Figure 3A and Figure 3B are heatmaps showing the strong correlation between the concentrations of transcription factors (*ACE2* and *SWI4*) and the expression levels of their direct targets linked to the hotspot. Our results are consistent with previous findings that suggest *ACE2* as a causal transcription factor mediating the global regulation of the mitotic-exit network (MEN) by *AMN1* [23] even though *ACE2*'s direct targets were not overrepresented for any GO biological processes or functional groups. This is probably because many downstream transcripts of the MEN were not considered in this analysis because there's no direct ChIP-Chip evidence of binding between these transcripts and *ACE2*.

Another interesting regulatory hotspot, occurring at chromosome 12 basepairs 1,040,000 to 1,060,000, was found by Brem et al. to regulate subtelomerically encoded helicases through the causal regulator *SIR3*. We found two transcription factors, *GAT3* and *YAP5*, whose concentrations were linked to this hotspot using the Harbison et al. data. *YAP5* was also significant using the Lee et al. data. Figure 3D and Figure 3C show the strong correlations between *GAT3* and *YAP5* concentrations and the expression profiles of their targets. Unlike the previous example, the targets of *YAP5* were enriched for helicases (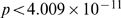) and consisted of many genes with unknown function as represented by a significant enrichment for the GO annotation of “biological process unknown” (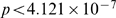). These results suggest a potential novel mechanism for the regulation of subtelomerically encoded helicases mediated by *YAP5* and *GAT3*.

### Regulatory Hotspots Perturbed the Promoter Affinities of Transcription Factors To Give Rise to Global Differential Expression

We next considered SNPs located in regulatory hotspots that perturbed the promoter affinities of transcription factors to cause global differential expression. Modeling these perturbations required an extension to the NCA model. As shown in Figure 2D, in addition to decomposing the transcription factor concentration and gene expression matrices, we also decomposed the promoter affinities matrix, 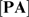 into 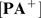 and 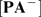 where the only difference between the two is the column corresponding to the global promoter affinities of the transcription factor of interest as shown in bold. We identified perturbed networks of genes and transcription factors by deriving a likelihood ratio statistic that compared the extended model to the original NCA model. Since the extended model included additional parameters, namely different promoter affinities between populations, we expected it to always fit the data better. Thus to assess significance, we used a permutation scheme that randomized the decomposition of individuals while preserved the topology of the bipartite graph (see Methods).

We revisited the regulatory hotspots discussed in the previous section. We speculated that transcription factors whose promoter affinities were perturbed by a regulatory hotspot must interact with other transcription factors whose concentrations were perturbed by the same hotspot to induce global differential expression of the targets. The intuition being if the *in vivo* concentrations of a transcription factor is relatively stable, then it could still regulate gene expression by differentially binding to other transcription factors to form a complex. A transcription factor's binding affinity for promoters is then in part determined by the concentrations of its partnering transcription factors. This is exactly what we observed in our results. For example, we found that hotspot 6 which was shown to be linked to the concentrations of *HAP1* was also linked to the promoter affinities of *HAP4*. *HAP1* and *HAP4* are known to interact in a complex to regulate global respiratory gene expression. Similarly, hotspot 8 was linked to the concentrations of *DIG1* and the promoter affinities of *STE12*. *DIG1* has previously been shown to code for an inhibitor of *STE12*, a transcription factor involved in pheromone induction and invasive growth [32]–[34]

**Figure 4:**
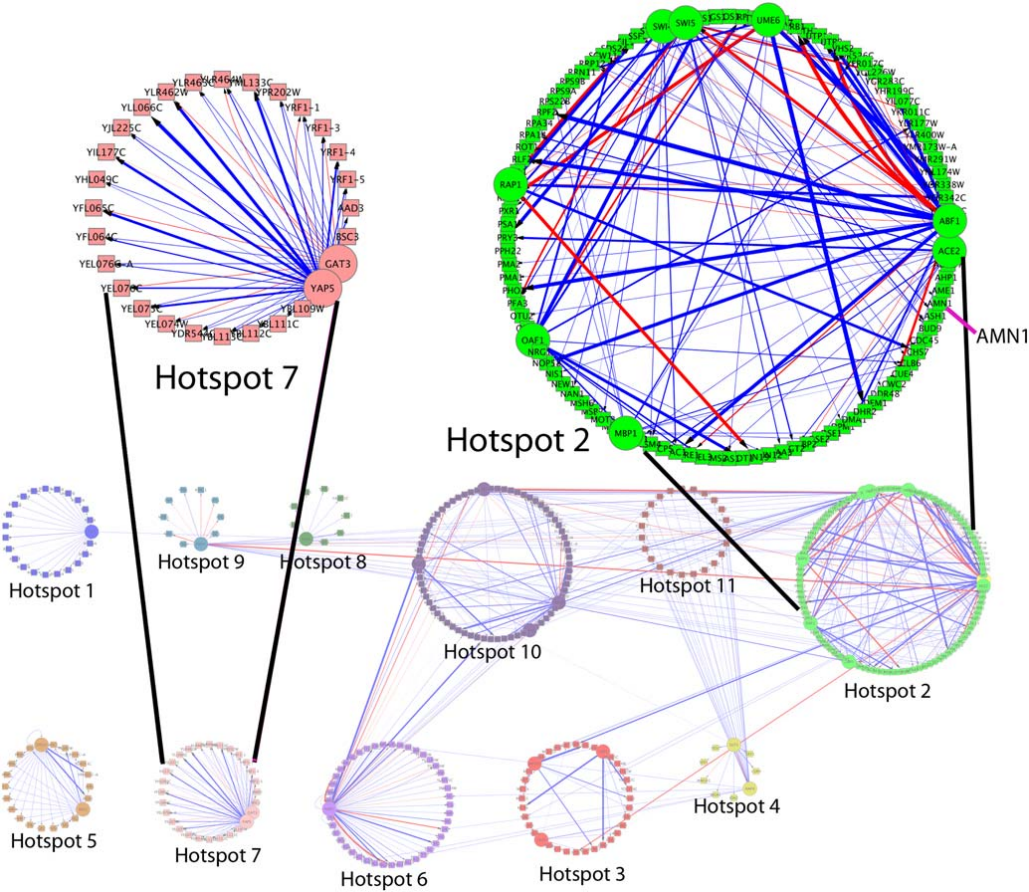
Networks perturbed by regulatory hotspots. Eleven hotspots and the networks of transcription factors and target genes perturbed. Large circular nodes represent transcription factors and square nodes represent target genes. The thickness of an edge represents how much a hotspot perturbs the promoter affinity. Red edges designate a change of a transcription factor from an activator to a repressor or vice versa. Notice that some perturbed networks share transcription factors. We show two hotspots and the corresponding networks in detail. Hotspot 2 in addition to affecting the promoter affinities of *ACE2* and *SWI4*, also affects the promoter affinities of several other transcription factors, including *UME6*, which is known to interact with *ACE2*. Hotspot 7 affects the promoter affinities of *YAP5* (thick edges) but its affect on *GAT3* promoter affinities is not statistically significant (thin edges). Figure was generated using the Cytoscape software [42].

We next examined how two hotspots discussed in the previous section also perturbed promoter affinities of transcription factors. Figure 4 and Table 2 show that hotspot 2 was linked to the promoter affinities of *ACE2*, *SWI4* and *UME6*. Hotspot 2 was also shown in the previous section to be linked to the concentrations of *ACE2* and *SWI4* but not *UME6*, see Figure S2 for the expression profiles of the downstream targets of *UME6*. Consistent with our speculation, *UME6* has been shown to interact with *SWI4* and *SWI4* has been shown to interact with itself. Furthermore, we see that *AMN1* is a target of *ACE2* suggesting that the regulation of the mitotic-exit network might be feedback in nature.

**Table 2:** Regulatory hotspots and the transcription factors whose promoter affinities are perturbed to achieve global regulation.

	Hotspot Location			# Linkages		Significant Transcription Factors		
	Chr	Begin	End	Lee	Harbison	Lee	Harbison	Shared
1	2	360000	380000	24	29	None	None	None
2	2	480000	580000	103	142	None	SWI4, ACE2, UME6	None
3	3	60000	100000	89	113	MET31	ABF1	None
4	5	340000	440000	34	48	None	None	None
5	8	80000	120000	36	51	None	STE12	None
6	12	600000	680000	54	91	None	HAP4	None
7	12	1040000	1060000	8	12	MSN4	None	YAP5
8	13	40000	60000	20	27	None	None	None
9	14	440000	500000	130	179	ABF1	None	FKH1
10	15	140000	200000	76	117	RAP1	HAP1, SKN7	SWI4, CIN5
11	15	560000	580000	21	26	None	None	None

Figure 4 also shows a similar network consisting of the two transcription factors whose concentrations linked to hotspot 7, *GAT3* and *YAP5*. Notice that while *YAP5*'s promoter affinities were linked to the hotspot also (thick edges), *GAT3*'s were not (thin edges). Consistent with previous results, *YAP5* has been shown to interact with itself to modulate gene expression. These results suggest that in some transcription factors, particularly those that interact with themselves, both promoter affinities and concentrations of the transcription factor could be perturbed by a regulatory hotspot. On the other hand, some transcription factors might not have their concentrations perturbed by a hotspot but because of interactions with another transcription factor, has their promoter affinities perturbed giving rise to global differential expression of their targets.

### Most *cis* SNPs Perturbed the Local Promoter Affinities of Target Genes

Previous eQTL analyses have shown that the most significant linkages occur *cis* to genes [1],[2]and often located or in LD with SNPs located in the promoter regions of genes harboring transcription factor binding sites [35]. Our model allowed us to determined if differences in expression of a single gene could be attributed to *cis* genetic variations perturbing the local affinities of transcription factors on the promoter.

There is a direct similarity between these perturbations and those that affect global promoter affinities. As shown in Figure 2E, SNP3 perturbs the local affinities of transcription factors for the promoter of G3. We modeled this affect by decomposing the 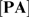 matrix into 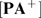 and 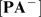 where the only difference between the decomposed matrices was the row corresponding to G3, as shown in bold. We used a likelihood ratio statistic to choose between two different models and assessed the significance based on permuting the genotypes of the individuals.

Of the small subset of genes examined, 2294 from using the Lee et al. dataset and 2779 from using the Harbison et al. dataset, we found ≈45% of the transcripts (972/2294 Lee, 1315/2779 Harbison) linked to at least one SNP at a FDR of 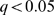 with 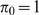 using a standard *t*-test. Out of these linkages, ≈30% were *cis* (257/972 Lee, 331/1315 Harbison). These proportions are consistent with what has been reported [10].

We postulated that many *cis* linked loci found by previous analyses and confirmed by our analysis are in LD with causal SNPs located in promoter regions. We further postulated that such a causal SNP corresponds to a variation in the primary sequence of a transcription factor binding site that affects the promoter affinity of a transcription factor or a complex of transcription factors. This model is consistent with the idea that a genetic variation at regulatory regions of the genome can give rise to observed subtle differences in gene expression across populations. We identified a total of 138 and 174 genes which have their local promoter affinities affected by a SNP with a FDR of 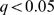.

**Figure 5:**
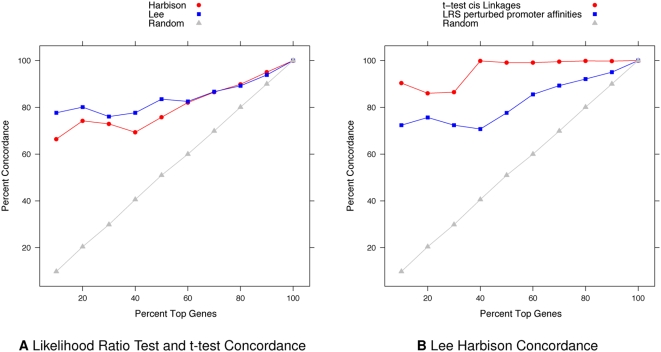
Concordances between applying different statistical tests and using different protein-DNA binding datasets. (A) Percent of top genes with promoter affinities perturbed detected by likelihood ratio test concordant with those with *cis* linkages detected by t-test (red: Harbison dataset, blue: Lee dataset, gray: random). (B) Percent of top genes concordant between Lee and Harbison datasets using different tests (red: t-test for *cis* linkages, blue: likelihood ratio test for perturbed promoter affinities, gray: random).

Figure 5A shows that there is high concordance between those genes with significant *cis* linkages and those whose promoter affinities were perturbed. We did not expect all *cis* linkages to perturb promoter affinities. There are potentially other regulatory machinery that operate on intronic 3′UTRs and 5′UTRs. Next we compared the perturbed genes found using the Lee et al. dataset versus those found using the Harbison et al. dataset (Figure 5B). At a FDR of 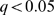, 72 significant genes were shared between the datasets and 168 genes were not. We suspected that the different results obtained from these two datasets can be attributed to differences in network topology. The two binding datasets often reported genes with different sets of bound transcription factors and transcription factors with different sets of targets making the estimates of certain quantities inconsistent. Additional discrepancies arose from different sets of genes having been eliminated from each analysis due to the criteria placed on the network topology.

## Discussion

Although there is a growing wealth of literature identifying putative causal regulators in yeast and mouse using statistical approaches, some of which integrate different sources of information, it is not clear by what mechanism genetic variations perturb the underlying regulatory networks to give rise to global differential expression. We have presented an integrated framework based on network component analysis that directly models how genetic variations perturb the concentrations and promoter affinities of transcription factors to cause the differential expression of their targets. Such a model differs from current eQTL analyses in that a direct, testable mechanism of transcription regulation is specifically considered. Although these networks are limiting, both in terms of the amount of biology they explain as well as the dependence on experimental data for their inference, a substantial set of genes (≈1/3) was still considered. In our analysis, we show that many genes with *cis* linkages are likely to be regulated by transcription factors binding differentially to their promoter regions. We also show two representative examples of the complex mechanism of achieving global differential expression of a large number of transcripts, where the regulation of transcription factors involve two distinct processes and maybe feedback in nature.

Our approach specifically uses one variation of the NCA algorithm to infer the concentrations and promoter affinities of transcription factors. The key aspect of our approach is that we treat genetic variations as perturbations to an underlying regulatory network whose structure is already known. In theory, any NCA like approach [36]–[38] where a network is inferred from known data such as ChIP-Chip experiments, protein-protein interaction experiments or literature can be extended to take into account genetic variation.

There are also some natural extensions to the framework we have presented. First, one is not limited to considering only genetic variation as a perturbation. Other forms of perturbation such as media condition and disease pathogenesis can as well be applied in this approach to identify the corresponding effect on the networks. Second, our method considers the perturbation of only one SNP. Although several approaches have been proposed to investigate the statistical interaction of multiple SNPs on a phenotype [11],[39], it would be interesting to study the mechanistic interactions of multiple perturbations on a transcription regulatory network.

## Methods

### Strains, Expression Measurements, and Genotyping

We used the expression measurements (6,216 transcripts) and genotyping data (2,956 SNPs) collected over 112 segregants of yeast derived from two parental strains BY4716 and RM11-1a originally described by Brem et al. The gene expression data is available at GEO (http://www.ncbi.nlm.nih.gov/projects/geo/) with the accession number GSE1990.

### Constructing Transcription Regulatory Networks from ChIP-Chip Data

ChIP-Chip data from two datasets [27],[28] were used to generate two different transcription regulatory networks at a *p*-value cutoff of 0.001. Consistency was checked in each case by comparing the networks generated from using a 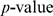 cutoff of 0.01 and 0.001.

We next checked for NCA compliance as outlined [31]. We were left with a sub-network of 2,294 transcripts and 100 transcription factors after processing the Lee et al. dataset and 2,779 transcripts and 158 transcription factors after processing the Harbison et al. dataset.

### Computing Genetic Linkage and Identifying Regulatory Hotspots

We first performed a standard *t*-test to compare the population means between the segregated expression profiles of a single gene by a given SNP. We assessed the significance of our linkages by performing a permutation test as described [40].

We then identified regulatory hotspots by dividing the yeast genome into 493 20 kb bins and counted the number of significant *trans* linkages to unique gene expression levels each bin contained from the standard *t*-test. We found a total of 430 significant *trans* linkages using the Harbison et al. data and 290 using the Lee et al. data. Assuming a Poisson process where the rare event of a *trans* linkage occurs at a rate of 0.87 (430/493 Harbison) and 0.60 (290/493 Lee), the probability of observing >7 linkages in the largest bin using the harbison_transcriptional_2004 data is 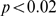 and the probability of observing >6 linkages in the largest bin using the Lee et al. data is 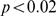. Because of the differences in the set of genes used in the different datasets, we constructed a set of 11 hotspots shared between the two.

### Application of NCA to Gene Expression Data Collected over a Population

NCA was originally developed to analyze time series based gene expression data but can be easily adapted to analyze whole genome expression data collected from different individuals in a population. In both cases, the goal is to infer the concentrations of active transcription factors and the promoter affinities from the expression levels of the target genes. This inference is made possible when the partial topology of the interaction network between transcription factors and target genes is determined from genome-wide location analysis that detects the binding of transcription factors to DNA promoter regions (ChIP-Chip).

Figure 1B shows an example of a bipartite graph where the expression levels of five genes are determined by the concentrations and promoter affinities of the three transcription factors. Formally, given a matrix 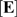 of dimension 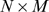 where we have collected the expression levels of 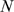 genes from 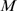 individuals. Each column 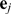 represents a separate microarray experiment that measures the expression levels of all genes in one individual. NCA approximates the relationship between the concentrations of active transcription factors and gene expression levels by a log-linear model of the type:
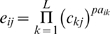
(1)where 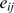 is the gene expression level for gene 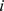 in individual 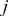, 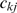 is the concentration of transcription factor 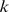 in individual 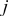 and 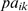 is the affinity of transcription factor 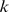 for the promoter of gene 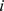. We can take the log of Equation 1 and transform it into a matrix representation:
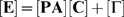
(2)Here, 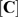 is a matrix of dimension 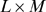 representing the concentrations of the 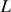 transcription factors in the 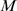 individuals and 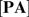 is a matrix of dimension 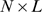 representing the affinities of the 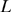 transcription factors for the promoters of the 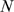 genes and 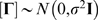 is a matrix of dimension 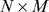 representing the residual. NCA analysis without incorporating genetic information seeks to iteratively find 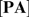 and 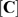 that minimizes the quantity:
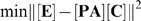
(3)Finding the least squares estimates of 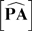 and 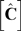 is equivalent to finding the maximum likelihood estimates under the assumption that the 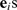 are independent identically-distributed (iid) vectors with Gaussian noise.

### Incorporating Genetic Variation into the NCA Model

In our model, a genetic variation induces global differential expression either by perturbing the concentrations of a transcription factor or the promoter affinities of a transcription factor on *all* of its targets. Figure 1C shows the former case where the promoter affinities of TF1 on all targets remain the same but the concentration of TF1 is elevated in the group of individuals with an A allele at SNP1 while it is attenuated in the group of individuals with the C allele at SNP1. Figure 1D shows the latter case where the affinities of TF2 for the promoter region of its targets are different between two populations. Notice that in both cases, we do not make any assumptions about where the genetic variation occurs since several mechanisms can contribute to the transcription factor having different *in vivo* concentrations and promoter affinities. We can formally model perturbations to the promoter affinities by constructing two matrices, 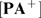 and 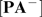 that differ in the column corresponding to the transcription factor of interest.

We can also model local changes to the promoter affinities of all transcription factors on a single gene such as shown in Figure 1E where one group of individuals has the A allele and another group has the T allele (SNP3) in the binding site of the transcription factor complex. To model this change in the promoter affinities on one gene, we again construct two matrices 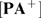 and 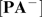 that differ in the row corresponding to the gene of interest.

#### Extending the NCA model to incorporate genetic perturbations

We can rewrite Equation 2 to incorporate perturbations on the promoter affinities:
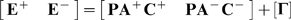
(4)where we have decomposed 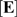 into 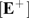 and 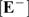, and 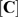 into 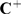 and 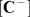 representing the expression levels and the inferred concentrations of transcription factors in two different populations segregated by a genetic variation. 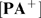 and 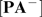 are the corresponding promoter affinity matrices of the two populations. 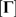 is again the residual.

#### Computing the linkage between transcription factor concentrations and genetic variations

If a genetic variation affects the concentrations of the transcription factors to induce differential expression, we can model the effect by decomposing the originally inferred 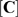 matrix into 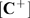 and 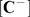 that differ in the row corresponding to the transcription of interest. For each transcription factor, we can then apply a simple *t*-test treating the concentration as a quantitative trait segregated by the genetic variation. We assess the significance of the statistic by shuffling the genotypes of the individuals 1000 times [40] and computing the false discovery rate (FDR) [41].

#### Computing the linkage between promoter activities and genetic variation using a likelihood ratio based statistic

Notice that if a genetic variation perturbs the promoter affinities either globally or locally, we can't simply compare the 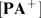 and 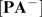 matrices. Instead, we can use model selection techniques to compare our more complex model with the simpler NCA model. Specifically, we define the optimization problem similar to Equation 3:
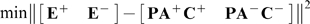
(5)We can approximate the solution to Equation 5 by running the original NCA algorithm and fixing the 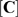 matrix and re-estimating the 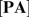 matrix.

To test the validity of our model, we define the null and alternative hypotheses corresponding to the two models as:

Hypothesis *H*_1_: The expression levels 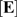 can be decomposed into 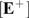 and 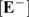 for those individuals with the major and minor alleles respectively; and approximated by a log-linear models characterized by the parameters 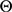:


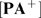
: A 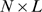 matrix representing the promoter affinities of transcription factors in individuals with the major allele


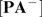
: A 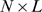 matrix representing the promoter affinities of transcription factors in individuals with the minor allele


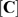
: A 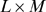 matrix that can be decomposed into 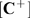 and 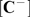 representing the concentrations of active transcription factors in the 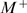 individuals with the major allele and 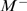 individuals with the minor allele respectively.

Hypothesis *H*_0_: The expression levels 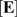 can be approximated by a log-linear model characterized by the parameters 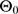:


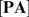
: A 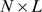 matrix representing the promoter affinities of transcription factors in all individuals (i.e. 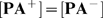).


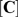
: A 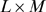 matrix representing the concentrations of transcription factors in all individuals.

When a genetic variation perturbs the promoter affinities to one gene locally, the difference in the number of parameters is equal to the number of regulators of the target gene. If we are re-estimating the promoter affinities globally for one transcription factor, the difference in the number of parameters is equal to the number of targets of the transcription factor. In both cases, we can compare our alternative model against the null model using a likelihood ratio statistic remembering that the 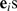 are independent.
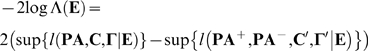
(6)

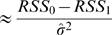
(7)

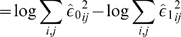
(8)where 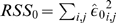 and 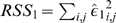 are the residual sum of squares from solving the least squares equations for 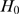 and 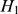 respectively. We estimate the two error variances 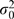 and 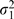 from the residual sum of squares of the larger model:
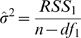
(9)where 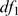 is the degrees of freedom of the model.

The above statistic follows the 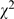 distribution asymptotically. However, since we are not re-estimating the full model in our extension, we perform permutations by rearranging the genotype labels of the individuals [40] 1000 times. We further estimated the significance of the permuted *p*-values by computing the false discovery rate [41].

## Supporting Information

Figure S1 Heatmaps showing correlations between concentrations of known transcription factors and the expressions of their targets. This figure shows heatmaps of the concentration levels of (A) *HAP1* and (B) *LEU3*, two transcription factors known to mediate global regulation, correlated with the expression levels of their downstream targets.
(0.40 MB TIF)

Figure S2 Heatmap showing lack of correlation between *UME6* concentrations and the expressions of its targets. This figure shows that *UME6*'s concentrations are not perturbed by regulatory hotspot 2 but the expression levels of its targets are.
(0.58 MB TIF)

Figure S3 Heatmaps showing correlation between transcription factor concentrations and expression levels. The heatmaps show the correlation between expression levels and concentrations of transcription factors for (A) 158 transcription factors in the Harbison dataset and (B) 100 transcription factors in the Lee dataset.
(0.68 MB TIF)

Figure S4 Heatmaps showing correlation of transcription factor concentrations between two datasets. A heatmap that shows the correlation of inferred transcription factor concentrations between the Harbison and Lee datasets.
(0.68 MB TIF)
