# Supplementary figures and images for: Using Network Component Analysis to Dissect Regulatory Networks Mediated by Transcription Factors in Yeast
Source: PLoS Comput Biol. 2009 Mar 20;5(3):e1000311. doi: 10.1371/journal.pcbi.1000311 (PMC2649002; doi:10.1371/journal.pcbi.1000311)

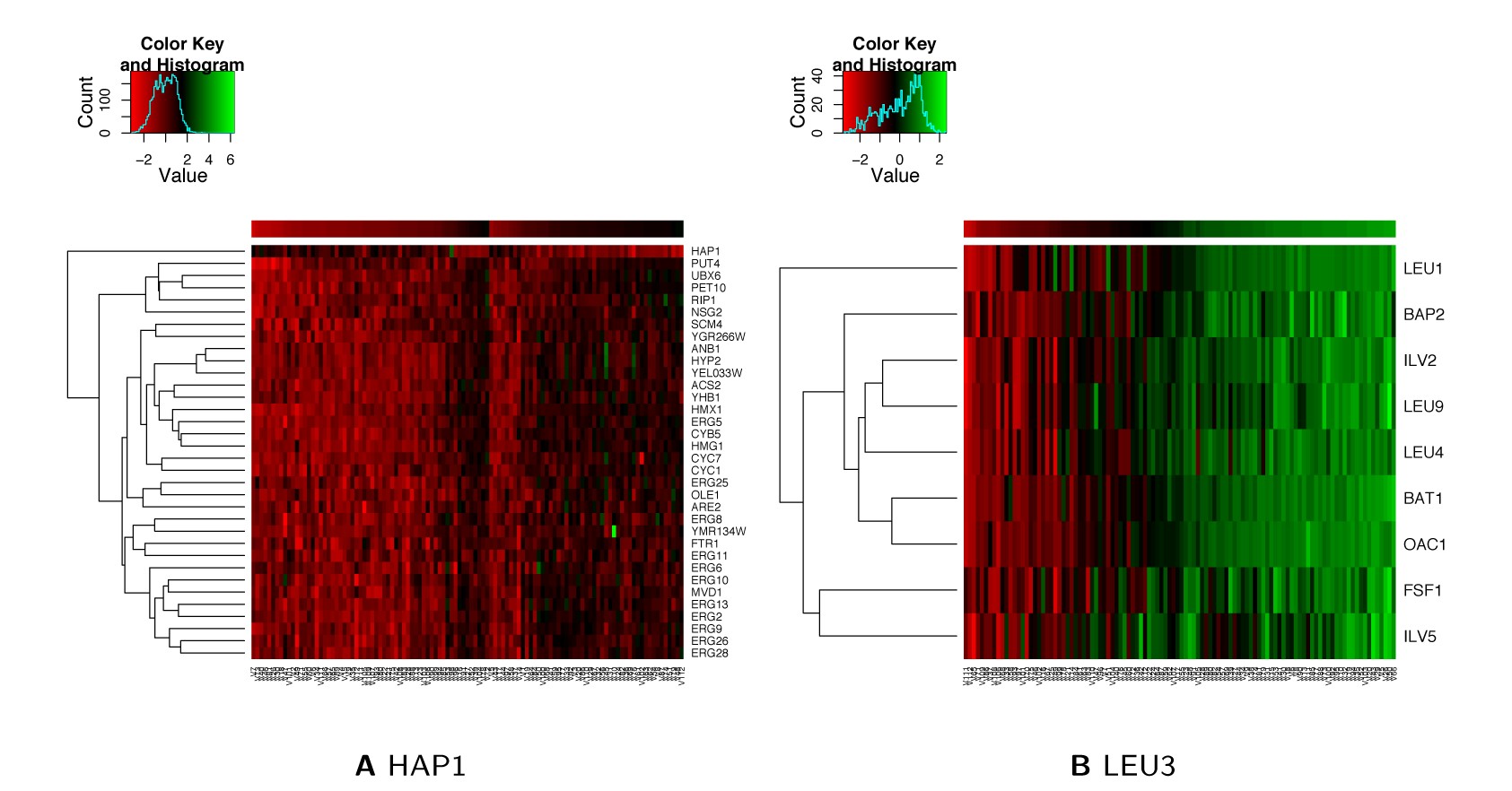

Supplement: Figure S1 — Heatmaps showing correlations between concentrations of known transcription factors and the expressions of their targets. This figure shows heatmaps of the concentration levels of (A) HAP1 and (B) LEU3, two transcription factors known to mediate global regulation, correlated with the expression levels of their downstream targets. (0.40 MB TIF) [file pcbi.1000311.s001.tif]

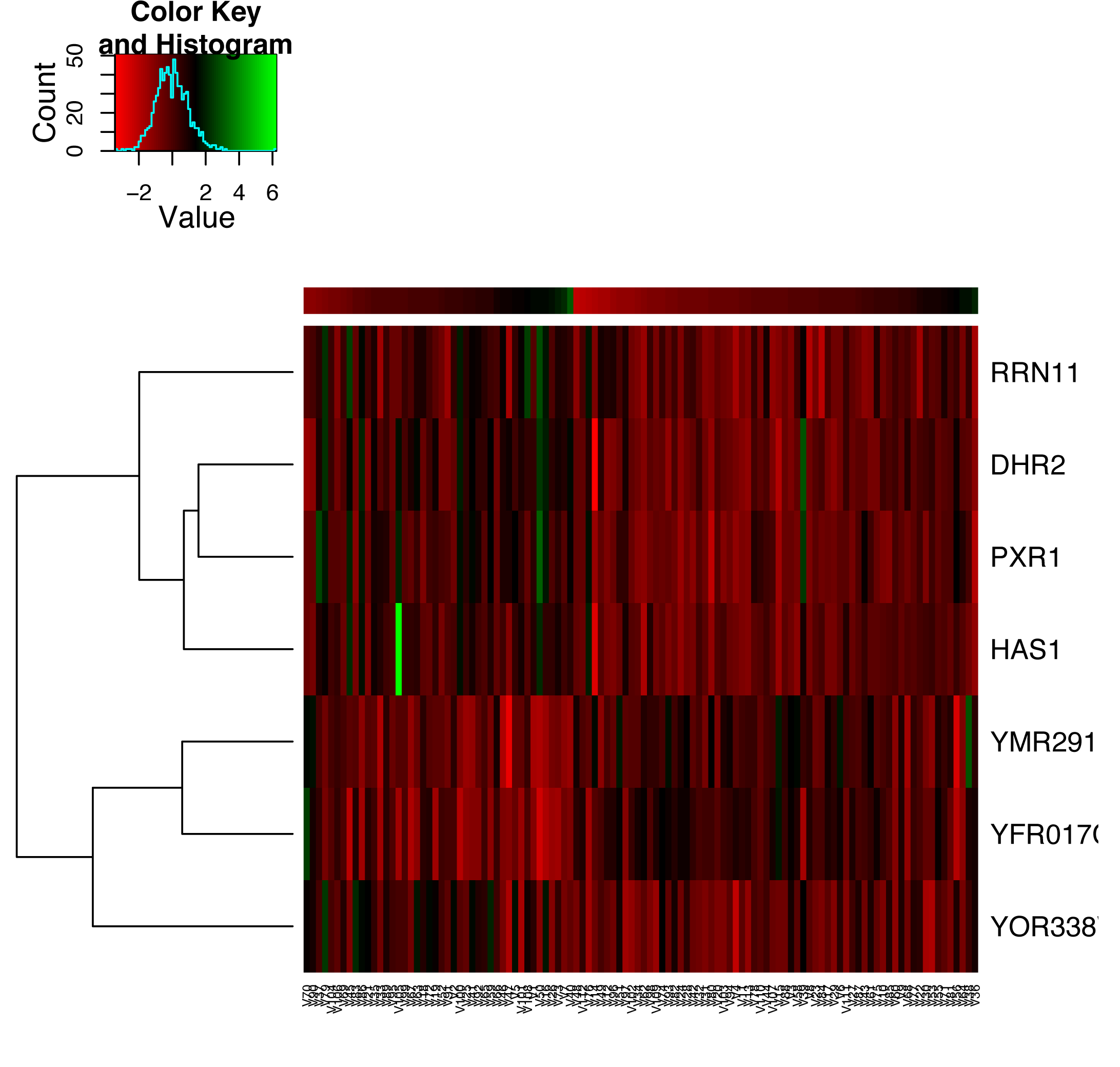

Supplement: Figure S2 — Heatmap showing lack of correlation between UME6 concentrations and the expressions of its targets. This figure shows that UME6's concentrations are not perturbed by regulatory hotspot 2 but the expression levels of its targets are. (0.58 MB TIF) [file pcbi.1000311.s002.tif]

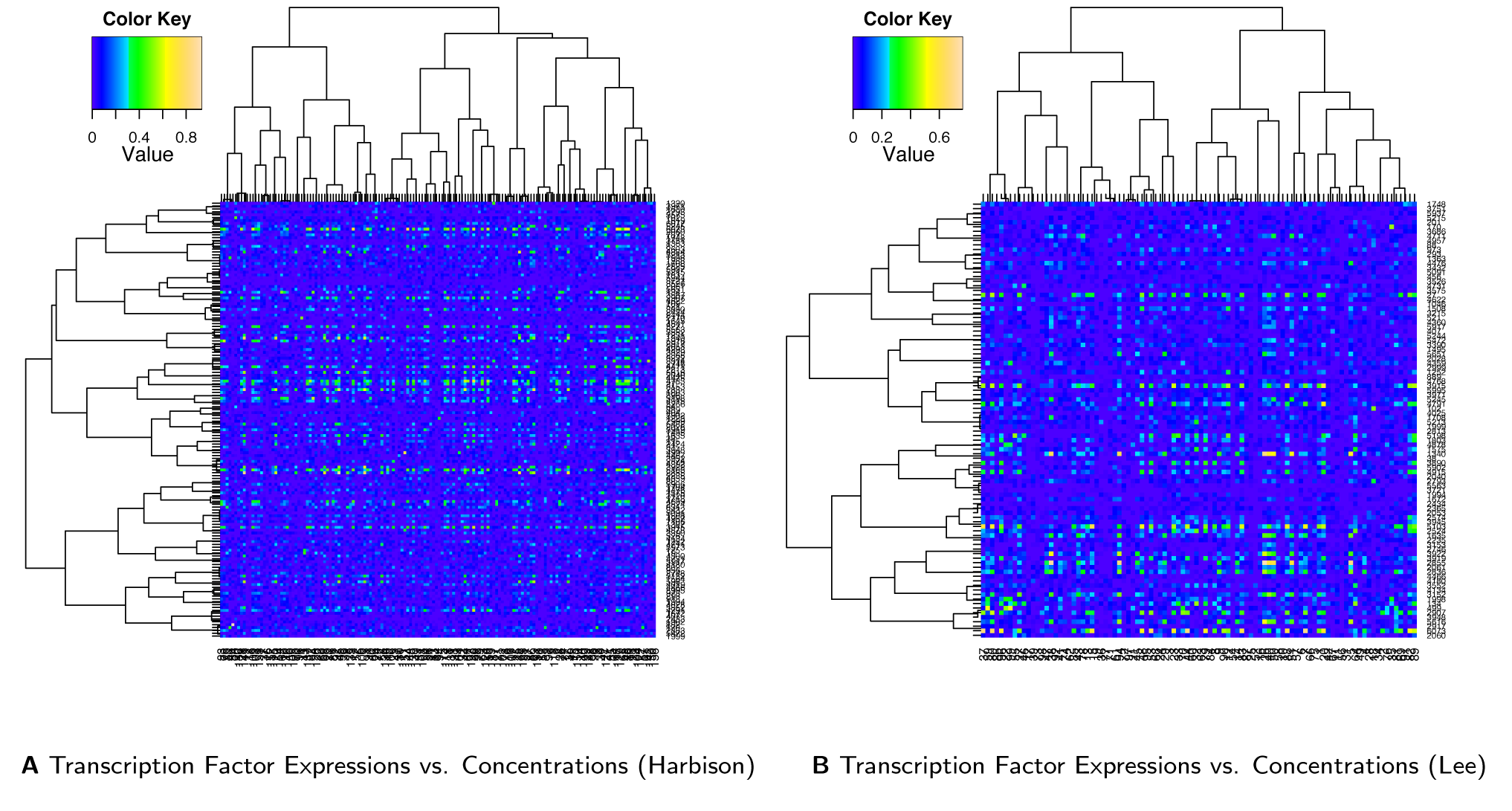

Supplement: Figure S3 — Heatmaps showing correlation between transcription factor concentrations and expression levels. The heatmaps show the correlation between expression levels and concentrations of transcription factors for (A) 158 transcription factors in the Harbison dataset and (B) 100 transcription factors in the Lee dataset. (0.68 MB TIF) [file pcbi.1000311.s003.tif]

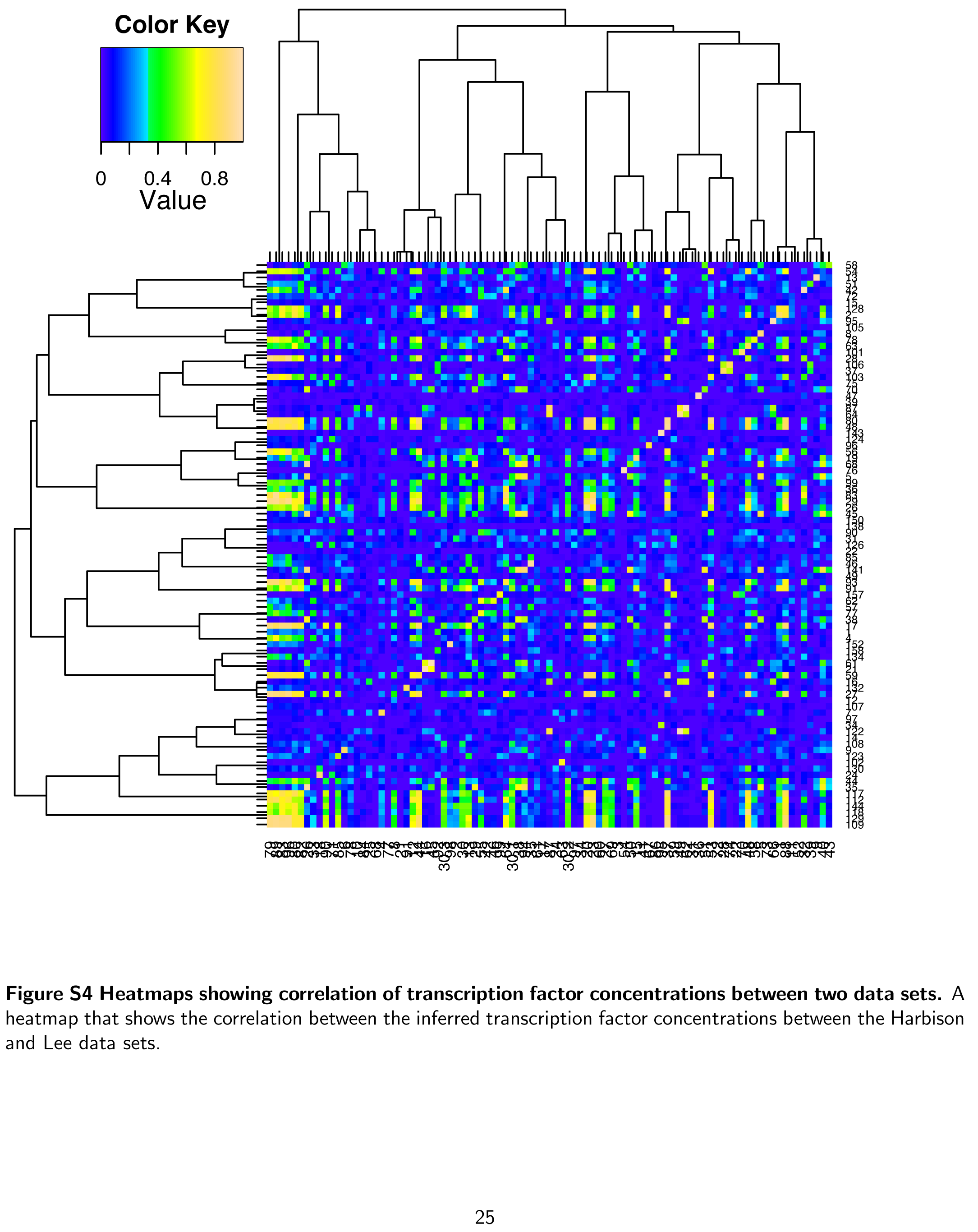

Supplement: Figure S4 — Heatmaps showing correlation of transcription factor concentrations between two datasets. A heatmap that shows the correlation of inferred transcription factor concentrations between the Harbison and Lee datasets. (0.68 MB TIF) [file pcbi.1000311.s004.tif]
